# Spatial distribution of physiologic 12-lead QRS complex

**DOI:** 10.1038/s41598-021-83378-8

**Published:** 2021-02-22

**Authors:** Katerina Hnatkova, Irena Andršová, Ondřej Toman, Peter Smetana, Katharina M. Huster, Martina Šišáková, Petra Barthel, Tomáš Novotný, Georg Schmidt, Marek Malik

**Affiliations:** 1 National Heart and Lung Institute, Imperial College, ICTEM, Hammersmith Campus, 72 Du Cane Road, Shepherd’s Bush, London, W12 0NN England; 2 Department of Internal Medicine and Cardiology, Faculty of Medicine, University Hospital Brno, Masaryk University, Jihlavská 20, 625 00 Brno, Czech Republic; 3 Wilhelminenspital der Stadt Wien, Montleartstraße 37, 1160 Vienna, Austria; 4 Klinikum Rechts der Isar, Technische Universität München, Ismaninger Straße 22, 81675 Munich, Germany

**Keywords:** Computational biology and bioinformatics, Physiology, Cardiology

## Abstract

The normal physiologic range of QRS complex duration spans between 80 and 125 ms with known differences between females and males which cannot be explained by the anatomical variations of heart sizes. To investigate the reasons for the sex differences as well as for the wide range of normal values, a technology is proposed based on the singular value decomposition and on the separation of different orthogonal components of the QRS complex. This allows classification of the proportions of different components representing the 3-dimensional representation of the electrocardiographic signal as well as classification of components that go beyond the 3-dimensional representation and that correspond to the degree of intricate convolutions of the depolarisation sequence. The technology was applied to 382,019 individual 10-s ECG samples recorded in 639 healthy subjects (311 females and 328 males) aged 33.8 ± 9.4 years. The analyses showed that QRS duration was mainly influenced by the proportions of the first two orthogonal components of the QRS complex. The first component demonstrated statistically significantly larger proportion of the total QRS power (expressed by the absolute area of the complex in all independent ECG leads) in females than in males (64.2 ± 11.6% vs 59.7 ± 11.9%, p < 0.00001—measured at resting heart rate of 60 beats per minute) while the second component demonstrated larger proportion of the QRS power in males compared to females (33.1 ± 11.9% vs 29.6 ± 11.4%, p < 0.001). The analysis also showed that the components attributable to localised depolarisation sequence abnormalities were significantly larger in males compared to females (2.85 ± 1.08% vs 2.42 ± 0.87%, p < 0.00001). In addition to the demonstration of the technology, the study concludes that the detailed convolution of the depolarisation waveform is individual, and that smoother and less intricate depolarisation propagation is the mechanism likely responsible for shorter QRS duration in females.

## Introduction

As well known, the physiologically normal adult QRS complex is 80–125 ms wide^1,2^. It has also been long understood^3^ that in addition to the heart size and to the ventricular myocardial mass^4^, the duration of the QRS complex is influenced by the myocyte geometry and layer orientation^5^, Purkinje fibre tree structure^6^, and depolarising ion currents^7^. It is also known that compared to males, females have shorter QRS complex^8,9^, and that QRS duration disparities exist between races^9,10^.

The electrophysiologic reasons for the sex and race differences are poorly understood but they cannot be explained by the differences in heart sizes and myocardial volume alone. In proportional terms, adult heart sizes are less variable compared to the QRS duration^11^ and while heart size reasonably correlates with body size^12^, the QRS duration does not^9^. This all potentially makes the broad range of the normal QRS complex durations somewhat unexpected.

The usual understanding of cardiac electrophysiology considers depolarisation sequence as a rather simple ion exchange reaction that propagates along myocardial fibre bands with practically uncontrolled cell to cell excitation transmission across gap junctions. The contribution of the differences in myocyte geometric composition and of the details in Purkinje fibre distribution must thus be rather substantial to explain the appreciable spread of myocardial depolarization duration. Considering these histology factors, the inter-subject differences in the QRS width have to be largely caused by heterogeneity in the propagation of the depolarization sequence including the anisotropic differences between longitudinal and cross-fibre excitation transmission combined with gap junction discontinuities^13,14^. At the same time, while showing substantial inter-subject differences, QRS complex duration is reasonably stable within each healthy subject, especially when considering repeated measurements at corresponding underlying heart rates^9,15^. Thus, the anisotropy of excitation propagation is likely to show similar intra-subject stability combined with appreciable inter-subject variability.

To allow clinical assessment of the myocardial depolarisation anisotropy and to permit investigation of its implication among clinically defined populations, a non-invasive method for the assessment of the spatial distribution of myocardial excitation waveforms is needed, preferably based on standard 12-lead electrocardiograms (ECG). Having this need in mind, we have tested the application of singular value decomposition (SVD)^16^ to the 12-lead QRS complex electrocardiograms. SVD was previously successfully used in a broad spectrum of electrocardiographic studies including, among others, ECG compression^17,18^, noise removal^19^, interval measurement^20^, and ECG component complexity analyses^21–23^. For the purposes of the present study, we have extended SVD algorithms as well as the interpretation of their results applied to the QRS signal complexity analysis. In addition to testing the application of the technology, we aimed at investigating whether the SVD signal analysis would help explaining the differences in QRS complex duration among healthy subjects and whether, similar to the differences in the QRS duration, it would show differences in QRS composition between the sexes. We have researched this using data that have previously been collected in a battery of clinical pharmacology studies.

## Methods

### Investigated population and electrocardiographic recordings

A collection of Holter recordings previously analysed for a different purpose was used^24^. Altogether 639 healthy subjects participated at six different clinical pharmacology studies. All subjects were screened before enrolment and all had a normal resting ECG and normal clinical investigation as mandated in clinical pharmacology research^25^. Clinical conduct of the studies including the procedures of electrocardiogram acquisition adhered strictly to the relevant guidelines and regulations^25,26^. All these studies were ethically approved by the institutional ethics bodies (Focus in Neuss; Parexel in Baltimore, Bloemfontein, and Glendale; PPD in Austin; and Spaulding in Milwaukee). All subjects gave informed written consent to the participation. All the source studies were conducted in accordance with the Helsinki declaration.

As previously described^24^, each of the studies included repeated 12-lead day-time Holter recordings in each participant. The recordings were made during multiple baseline days. During these baseline days, study protocols included repeated provocative manoeuvres with the aim of capturing wide heart rates ranges in each participant. The postural provocative manoeuvres included time-points during which the study subjects were, per protocol, in undisturbed supine, unsupported sitting, and unsupported standing positions. In addition to these, other study per-protocol time-points required the subjects to maintain strict supine positions. The Holter recordings used Mason-Likar electrode positions. Clinical conduct of the baseline days did not differ between individual units. The investigation described in this text utilized the baseline Holter recordings when the subjects were off any medication, did not smoke, and refrained from consuming caffeinated drinks. Further details of the clinical pharmacology studies are therefore irrelevant.

### Electrocardiographic measurements

Using previously developed technology combining computerized signal processing with visual checks and manual corrections of the measurements^24,27,28^, multiple 10-s segments were extracted from each of the Holter recordings aiming at the inclusion of segments with different underlying heart rates. That is, in addition to the extractions at pre-specified study time-points described in the previous section, instantaneous heart rate was measured throughout the complete day-time Holters and additional segments were extracted to cover the range of captured heart rates uniformly. These additional extractions were made during times when the study subjects were not restricted by study protocols although the standard clinical pharmacology research requirements were observed^20^. Apart from the postural provocative manoeuvres, the source studies did not include any physical or mental challenges. During the day-time Holter recordings, the subjects were allowed neither to sleep nor to leave the confined area of the clinical unit.

For each extracted segment, 2-min history of preceding RR interval was obtained and within these 2-min intervals, heart rate was measured in non-overlapping 30-s sections. Extracted segments were used in this study only if they were preceded by reasonably stable heart rates which were defined as the range between minimum to maximum heart rate measured in the 30-s sections not exceeding 5 beats per minute (bpm).

Each extracted 10-s ECG segment was filtered to reduce noise pollution and to eliminate baseline wander^27,28^. Subsequently, a representative median beat was constructed, as previously used in other ECG studies^28,29^. Specifically, in the waveforms of the original recording sampled at 1000 Hz, QRS complexes were identified, superimposed by obtaining autocorrelation maxima across all independent leads, and sample by sample medians of all superimposed P-QRS-T morphologies were used to form the representative beat (our unpublished experience suggests that by eliminating outlier values, the sample-by-sample medians reduce the noise of the representative beat compared to sample-by-sample averages). In this representative beat, all 12 leads were superimposed on the same isoelectric axis and QRS onset and offset points were identified using previously developed algorithms^27,28^. The quality control of the identification of these points included visual verification and manual correction of computerized measurements by at least two independently working cardiologists with subsequent independent reconciliation in case of measurement disagreement. Pattern matching algorithms^30^ were also applied to ensure that comparable morphologies of QRS onset and offset were measured systematically.

In each measured ECG segment, the QRS width was defined as the time distance between the QRS onset and offset. The underlying heart rate was defined as the heart rate calculated from the averaged duration of the RR intervals in the preceding 2-min (which also included the 10 s of the measured ECG segment itself).

### Singular value decomposition

The SVD principles of ECG signal were published in detail many times before^16,20,22^. In brief, the 12-lead ECG contains only 8 algebraically independent leads I, II, V1, V2, …, V6 since the unipolar limb leads are only simple algebraic combinations of leads I and II. Hence, the ECG signal may be considered to constitute a matrix ${\mathbb{M}}^{8,{n}}$ of voltage values which has 8 rows ${\mathbb{m}}_{i}$ corresponding to individual leads, and ${n}$ columns, each corresponding to one time-instant. That is, each row ${\mathbb{m}}_{i}$ is a function of time and the values ${\mathbb{m}}_{i}\left(t\right)$, where $0\le t<{n}$, create the image of the $i$-th lead of the original ECG recording. The decomposition is based on an algorithm that creates a diagonal matrix ${{\varvec{\Sigma}}}^{8,{n}}$ and matrices ${\mathbb{U}}^{\mathrm{8,8}}$ and ${\mathbb{V}}^{{n},{n}}$ such that ${\varvec{\Sigma}}={\mathbb{U}}^{T}{\mathbb{M}}{\mathbb{V}}$, which means ${\mathbb{M}}={\mathbb{U}}{\mathbb{W}}$, where ${\mathbb{W}}^{8,{n}}={{\varvec{\Sigma}}{\mathbb{V}}}^{T}.\Sigma$ is a diagonal matrix with non-zero values only in the left-most diagonal. These elements of ${\varvec{\Sigma}}$ (all > 0) are the eigenvalues ${\left\{{\sigma }_{i}\right\}}_{i=1}^{8}$ of the decomposition while the columns of matrices ${\mathbb{U}}$ and ${\mathbb{V}}$ are the left and right singular vectors. The rows of matrix ${\mathbb{W}}$ are the algebraically orthogonal components ${\left\{{\lambda }_{i}\right\}}_{i=1}^{8}$ of the decomposition.

### Orthogonal ECG signal reconstruction

In previous electrocardiographic SVD applications, the orthogonal components of matrix ${\mathbb{W}}$ were standardly sorted according to the corresponding values of eigenvalues. For the purposes of the present investigation, we used a different approach.

Original ECG signal in matrix ${\mathbb{M}}$ can be reconstructed using only subset of components of orthogonal signal matrix ${\mathbb{W}}$. Specifically, for any subset of the 8 orthogonal components (i.e. any selection of one, two, or more of the components ${\lambda }_{i}$), a matrix ${\mathbb{X}}^{8,{n}}$ can be created, for which the rows of the subset are the same as the corresponding rows of ${\mathbb{W}}$ while other rows contain zeros. The original matrix ${\mathbb{M}}$ can then be approximated by matrix ${\mathbb{N}}^{8,{n}}={\mathbb{U}}{\mathbb{X}}.$ Each of the 8 rows of matrix ${\mathbb{N}}$ correspond to the approximation of the corresponding ECG lead that originally constituted the matrix ${\mathbb{M}}$.

Various ways of quantifying the difference between ${\mathbb{M}}$ and ${\mathbb{N}}$ ECG signal can be proposed. In this study, we used the perhaps simplest possibility which was to calculate the area between the signals of individual leads of ${\mathbb{M}}$ and ${\mathbb{N}}$. That is for each lead $l\in \left\{\mathrm{I},\mathrm{II},\mathrm{V}1,\mathrm{V}2,\dots ,\mathrm{V}6\right\}$, we calculated the difference ${\Delta }_{l}$ between original lead ${\mathbb{m}}_{l}$ and its approximation ${\mathbb{n}}_{l}$ as ${\Delta }_{l}=\sum_{t=0}^{{n}-1}\left|{\mathbb{m}}_{l}\left(t\right)-{\mathbb{n}}_{l}\left(t\right)\right|$ and quantified the difference ${\varvec{\Delta}}$ between ${\mathbb{M}}$ and ${\mathbb{N}}$ as the average of all ${\Delta }_{l}$ (i.e. the average over different leads).

Obviously, if the selected subset of the 8 orthogonal components was empty (i.e. if no orthogonal components were used), the matrix ${\mathbb{N}}$ contained only zeros and all ${\Delta }_{l}=\sum_{t=0}^{{n}-1}\left|{\mathbb{m}}_{l}\left(t\right)\right|$.

This allowed to order the orthogonal components ${\left\{{\lambda }_{i}\right\}}_{i=1}^{8}$ according to their contribution to the original ECG signal. That is, we firstly selected a single component ${\lambda }_{1st}$ such that if only this component was used in matrix ${\mathbb{X}}$, the corresponding ${\mathbb{N}}$ to ${\mathbb{M}}$ difference ${\varvec{\Delta}}$ was the smallest among all single components ${\lambda }_{i}$. Subsequently, we selected a second component ${\lambda }_{2nd}$ such that if the matrix ${\mathbb{X}}$ was composed of components (${\lambda }_{1st} \bigoplus$ ${\lambda }_{2nd}$)—i.e. if the matrix ${\mathbb{X}}$ had only two non-zero rows, the corresponding ${\mathbb{N}}$ to ${\mathbb{M}}$ difference ${\varvec{\Delta}}$ was the smallest among all two component combinations (${\lambda }_{1st} \bigoplus$ ${\lambda }_{i}$), where ${\lambda }_{i}\ne {\lambda }_{1st}$. The same process was repeated and ${\lambda }_{3rd}$ was selected for minimum approximation difference based on (${\lambda }_{1st} \bigoplus$ ${\lambda }_{2nd} \bigoplus$ ${\lambda }_{3rd}$), and so on, up to the selection of the last ${\lambda }_{8th}$ orthogonal component.

This selection of $\lambda$ components results in a sequence of ${\mathbb{N}}$ to ${\mathbb{M}}$ differences ${\left\{{{\varvec{\Delta}}}_{i}\right\}}_{i=0}^{8}$ where ${{\varvec{\Delta}}}_{i}$ corresponds to the approximation signals ${\mathbb{N}}$ composed of the first $i$ components selected during the described selection process. Clearly ${{\varvec{\Delta}}}_{0}\ge {{\varvec{\Delta}}}_{1}\ge {{\varvec{\Delta}}}_{2}\ge \dots \ge {{\varvec{\Delta}}}_{8}=0$. The absolute contribution of the $i$-th component to the reconstruction of original ECG signal ${\mathbb{M}}$ is equal to ${{\varvec{\Delta}}}_{i-1}-{{\varvec{\Delta}}}_{i}$. Clearly, the value of this absolute contribution depends on the magnitude of the original ECG and cannot be directly used for comparisons of different ECGs. For that purpose, it is appropriate to consider the contribution of the $i$-th component in relative terms, i.e. as a value ${\nabla }_{i}={({\varvec{\Delta}}}_{i-1}-{{\varvec{\Delta}}}_{i})/{{\varvec{\Delta}}}_{0}$.

### Geometric interpretation

**Figure 1:**
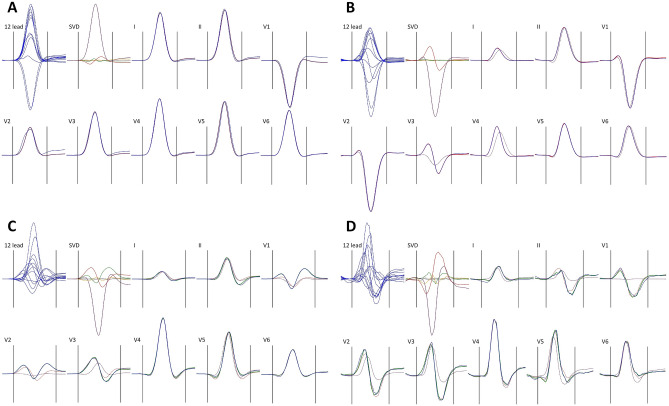
Examples of QRS reconstruction by orthogonal components. In all panels, superimposition of all 12 leads of the filtered representative median image of the QRS complex (12 lead) is shown together with the orthogonal components of matrix ${\mathbb{W}}$ (SVD) and with individual independent leads (I, II, V1, V2, …, V6) superimposed with the reconstruction. In all panels, the original ECG waveforms are shown in blue, the first, second, third, and fourth orthogonal components in violet, red, green, and amber, respectively (with all further components shown in grey). Panel (**A**) shows an ECG of a 51-year old female in which the reconstruction by the first component (full violet lines in the images of individual leads) explained 91.15% of the QRS area. Panel (**B**) shows an ECG of a 22-year old female in which the reconstruction by the first component (dotted violet lines in the lead images) explained 73.46% of the QRS area but the combination of the first and second component (full red lines in the lead images) explained 97.93% of the QRS area. Panel (**C**) shows an ECG of a 28-year old male in which the first and first + second component (dotted violet and red lines in the lead images, respectively) explained 58.49% and 72.56% of the QRS area but in which the combination of the first 3 components (full green lines in the lead images) explained 93.17% of the QRS area. Panel (**D**) shows an ECG of a 37-year old male in which the first, first + second, and first + second + third components (the same colour coding as in panel (**C**) explained 42.83%, 80.27%, and 87.76% of the QRS area, respectively.

When applying the SVD algorithm to the QRS complex, i.e. having the columns of matrix ${\mathbb{M}}$ spanning sample-by-sample between QRS onset and QRS offset, ${{\varvec{\Delta}}}_{0}$ becomes the absolute area under the QRS waveforms, i.e. the total “power” of the QRS signal (averaged over all 8 independent leads) and is measured in milliseconds*millivolts. The relative contribution of the first component ${\nabla }_{1}$ specifies the proportion of the signal that can be explained by projection of the depolarisation waveform only in one direction along (and backwards) of the main spatial QRS vector. There are only rare ECGs (example in Fig. 1A) in which almost all the QRS signal is explained by the first component, i.e. for which the electrocardiographic projection of the depolarisation waveform is practically only unidirectional. By the SVD principles, the second component corresponds to a projection of the depolarisation waveform that is perpendicular to the vector of the first component. That is, the sum ${\nabla }_{1}+{\nabla }_{2}$ specifies how much of the QRS signal can be explained by depolarization projection onto a 2-dimensional plane (defined by the perpendicular vectors of components ${\lambda }_{1st}$ and ${\lambda }_{2nd}$). In some ECGs in which the total QRS power is far from explained by the first component, projection onto a single plane explains most of the QRS signal (example in Fig. 1B). In other ECGs, even the sum of ${\nabla }_{1}+{\nabla }_{2}$ is still substantially below 1 and the third component needs to be added, i.e. more faithful reconstruction of the original signal is obtained by considering the electrocardiographic projection of depolarisation in 3-dimensional space (example in Fig. 1C). Nevertheless, once the QRS signal is fractionated differently in different leads, further components algebraically perpendicular to the 3-dimensional space of the first 3 components (well beyond mental spatial imagination) are needed to reconstruct the original signal (example in Fig. 1D).

For these reasons, the decomposition components ${\nabla }_{i}$ allow expressing the “proportion” of the ECG signal that is represented by electrical field changes in one vector direction, by changes along a combination of two perpendicular vectors, three perpendicular vectors, and so on. Similar attempts of quantifying these proportions were previously made based on decomposition eigenvalues ${\left\{{\sigma }_{i}\right\}}_{i=1}^{8}$^21,22,31^. To study the differences between these approaches, we applied the analysis to the QRS complex signals of the data analysed in this study and compared the ${\nabla }_{i}$ values with corresponding eigenvalue proportions  The comparison was based on their relative differences, i.e. values  which allowed to judge the comparisons independent of the dimension $i$.

### Covariates

**Figure 2:**
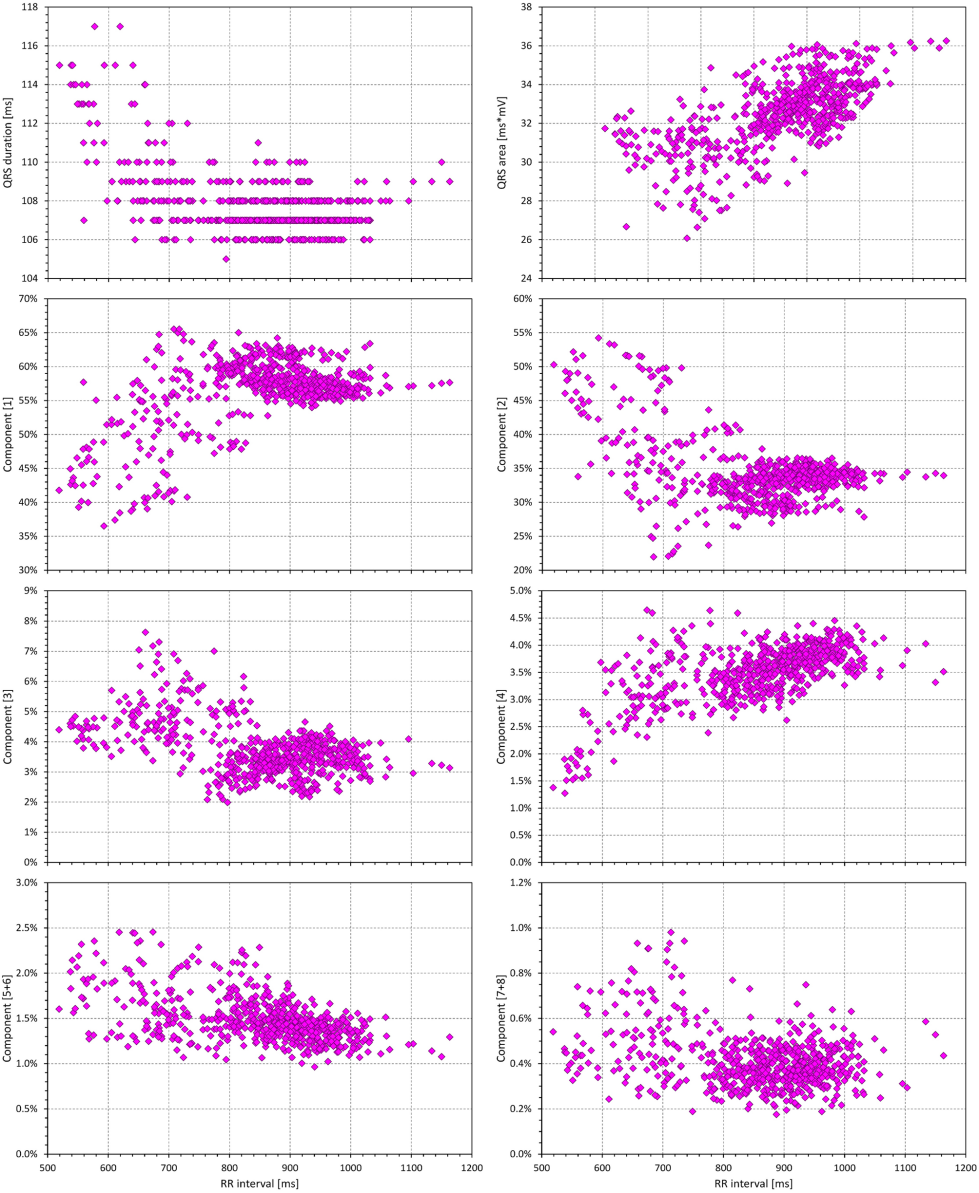
Example of relationship between values measured in a 23-year old male. Individual panels show the dependency of QRS duration, of absolute QRS area, and of selected orthogonal components and their combination on the RR intervals of the underlying heart rate.

**Figure 3:**
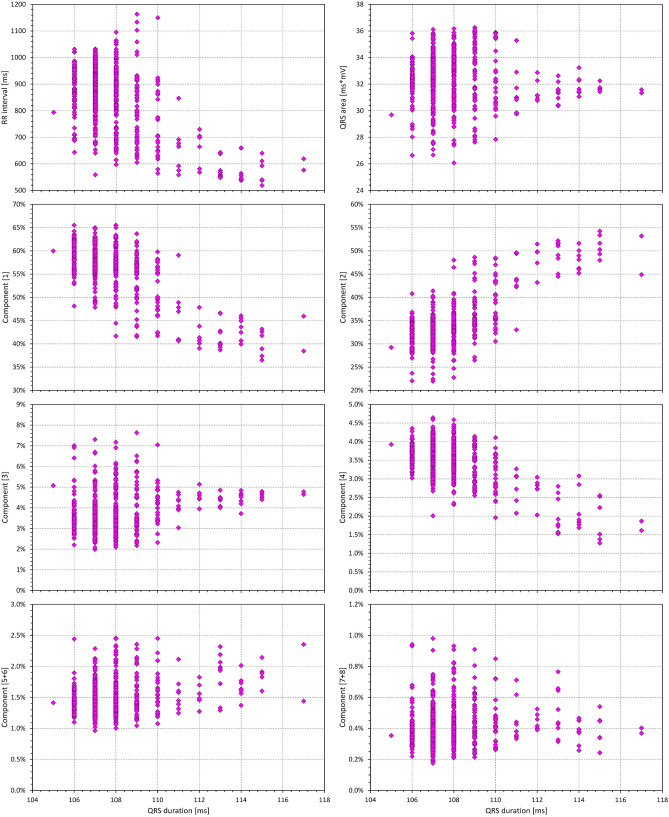
Example of relationship between measured values in the same 23-year old male as presented in Fig. 2. Individual panels show the dependency of absolute QRS area and of selected orthogonal components and their combination on the QRS duration. For completeness, the inverse relationship between the QRS duration and the RR interval of the underlying heart rate is also shown. The layout of the panels corresponds to that of Fig. 2.

In each study subject, covariate analysis was performed using data obtained from all ECGs of the given subject. The relative values of individual components as well as the values of their different combinations (i.e. values ${\nabla }_{i}+{\nabla }_{j}+\cdots$) were related to the RR intervals representative of the underlying heart rate of the corresponding ECG as well as to the duration of the QRS complex in the ECG. For this purpose, both linear and log-linear regression models were employed. As previously observed^9^, log-linear model fitted the relationship between QRS duration and the RR intervals better than the linear model. The intra-subject fits of the linear and log-linear models between the relative components and RR intervals and QRS duration were similar (i.e. for some of the components, linear model provided somewhat lower regression residua while for others, the fit by the log-linear model was tighter). For consistency, we have therefore used the log-linear regression models for all the intra-subject dependencies. Although some cases showed noticeable spread of the measured values (example in Figs. 2 and 3) the patterns of the regressions were still clearly detected.

To allow comparisons across subjects, these log-linear regression models were used to project the individual components and their combinations to the heart rate of 60 and 120 bpm, as well as to the QRS duration of 100 ms.

In each subject, Spearman correlation coefficients were also calculated between the values of the relative components and their combinations, and the RR intervals of the underlying heart rate, and the QRS durations.

### Statistics and data presentation

Data are presented as means ± standard deviation. Differences between subject groups (mainly between female and male participants) were evaluated using two-sample two-tail t-test assuming different variances of compared samples. Intra-subject comparisons (e.g. comparisons between components projected to heart rates of 60 and 120 bpm) were evaluated using two-tail paired t-test. P-values above 0.05 were considered statistically non-significant (NS). Because of the inter-dependency of evaluated data, no correction for multiplicity of testing was performed and all statistical tests performed are presented.

In each ECG, SVD calculations were made covering the interval between QRS onset and QRS offset. To avoid the possibility of the SVD results being influenced by QRS duration, the calculation was also repeated using a fixed width of the interval covered by matrix ${\mathbb{M}}$ in all ECGs. Specifically, this repeated calculation spanned the interval starting 5 ms before QRS onset and finishing 130 ms later. (The results of the fixed width calculations were only used to confirm the stability of the main analyses that are presented).

The SVD calculations were made using a custom software package developed in C++ (compiler of Microsoft Visual Studio version 15.4.0). Statistical evaluation used IBM SPSS package version 25.

## Results

### Population and electrocardiographic measurements

The source clinical pharmacology studies investigated 639 subjects (311 females). The ages of sex-defined sub-groups were practically identical (females 33.8 ± 10.1 years; males 33.9 ± 8.7 years, NS). The numbers of analysed ECG samples were also not different between females (609 ± 192) and males (591 ± 200, NS). The complete study evaluated 382,019 ECG samples.

The spread (i.e. the minimum to maximum range) of heart rates of the analysed ECG segments was also similar in females and males (57.8 ± 12.7 vs 56.2 ± 12.7 bpm, NS). Nevertheless, the spread of measured QRS widths was narrower in females (8.7 ± 5.8 ms) compared to that in males (10.2 ± 5.5 ms, p < 0.001). The regression-based extrapolation to the QRS width of 100 ms have thus involved some marginal extrapolations beyond available values, especially for females with markedly short QRS complexes.

**Figure 4:**
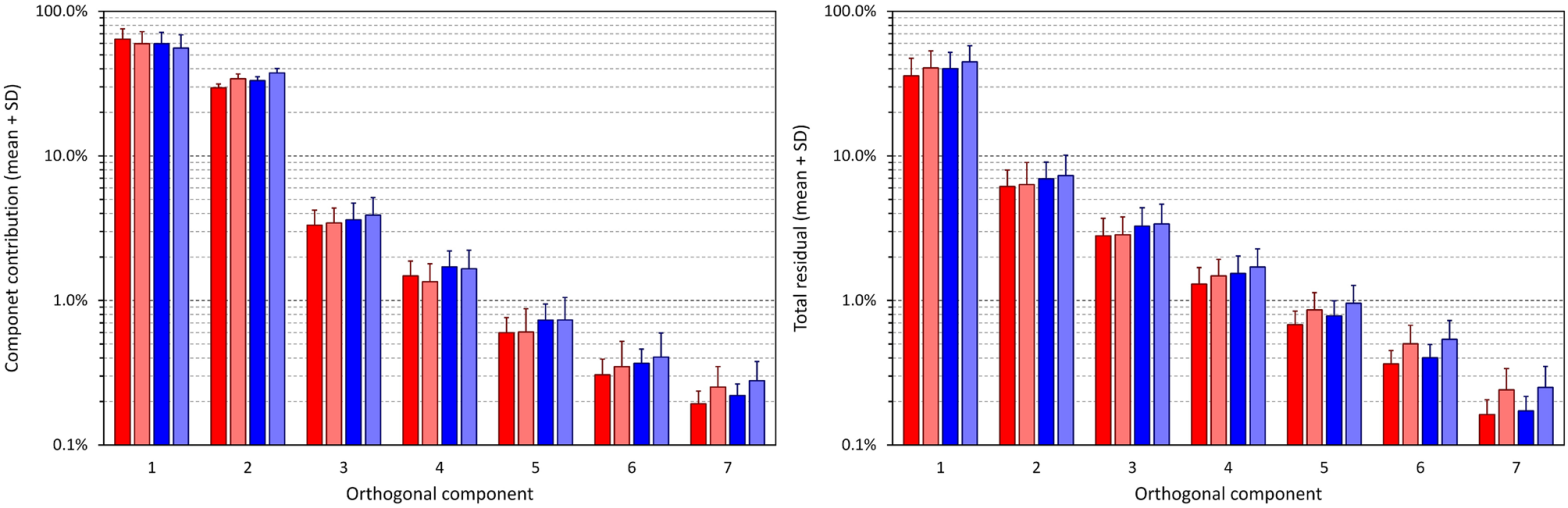
Summary of measured QRS components in individual study subjects. The right panel shows the contribution of individual components to the absolute QRS area, the left panel shows the proportion of the absolute QRS area remaining after the first, first + second, first + second + third, etc., component have been used to recompose the original electrocardiograms. The data in females and males are shown in red and blue, respectively, the dark and light columns correspond to the data projected to heart rates of 60 and 120 bpm, respectively. Mean values ± standard deviations are shown (note the logarithmic vertical axes). The 8th component is not shown since the combination of all 8 components covers the absolute QRS area fully and therefore, the contribution of the 8th component corresponds to the remainder after the use of the first 7 components.

Summaries of individual ECG measurements are shown Fig. 4, their numerical values are listed in the Supplementary Table. The observations made with the repeated SVD analyses using the fixed 130 ms intervals were practically the same (not shown here). That is, albeit the numerical values were little different, their proportions and sex comparisons led to the very same conclusions as described further.

### Comparison with eigenvalue proportions

**Figure 5:**
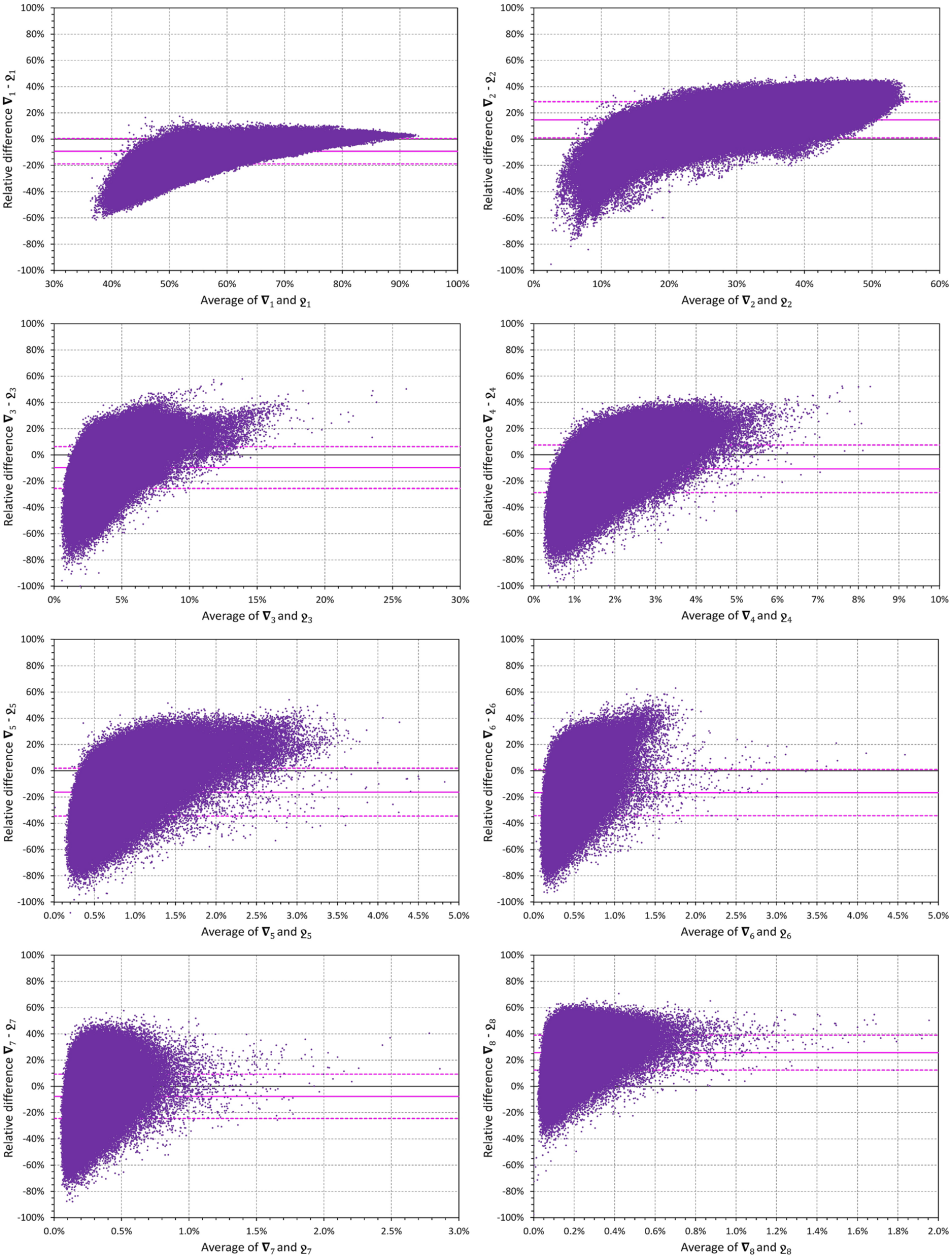
To demonstrate the differences between the decompositions approach that we used and the usual order of eigenvalues, the figure shows Bland–Altman type comparisons of relative eigenvalue proportions  and $\nabla_{i}$ for all $i$, $1 \le i \le 8.$ To allow comparison between different components, the vertical values show relative differences, that is values  The scatter diagrams show the data of all evaluated 10-s ECG samples, the bold horizontal lines are the means of the relative  values, the dashed lines are their means ± standard deviations. Note that the individual data include multiple readings in different subjects and that the values shown in the scatter diagrams are not fully mutually independent.

In 8.27% of the analysed ECG samples, the order of the decomposition components ${\nabla }_{i}$ was different from the order of SVD eigenvalues ${\sigma }_{i}$ that are standardly used for component ordering^16^. In 1.40% and 4.10% of the samples, this difference of the order occurred in the first 3 and in the 4th to 6th dimensions, respectively. More important, however, were the numerical differences between components ${\nabla }_{i}$ and the corresponding eigenvalue proportions  . These are displayed in Fig. 5 which shows that with higher dimensions, these numerical differences spanned between approximately − 80 to + 40%.

### Absolute area under the QRS waveform

**Figure 6:**
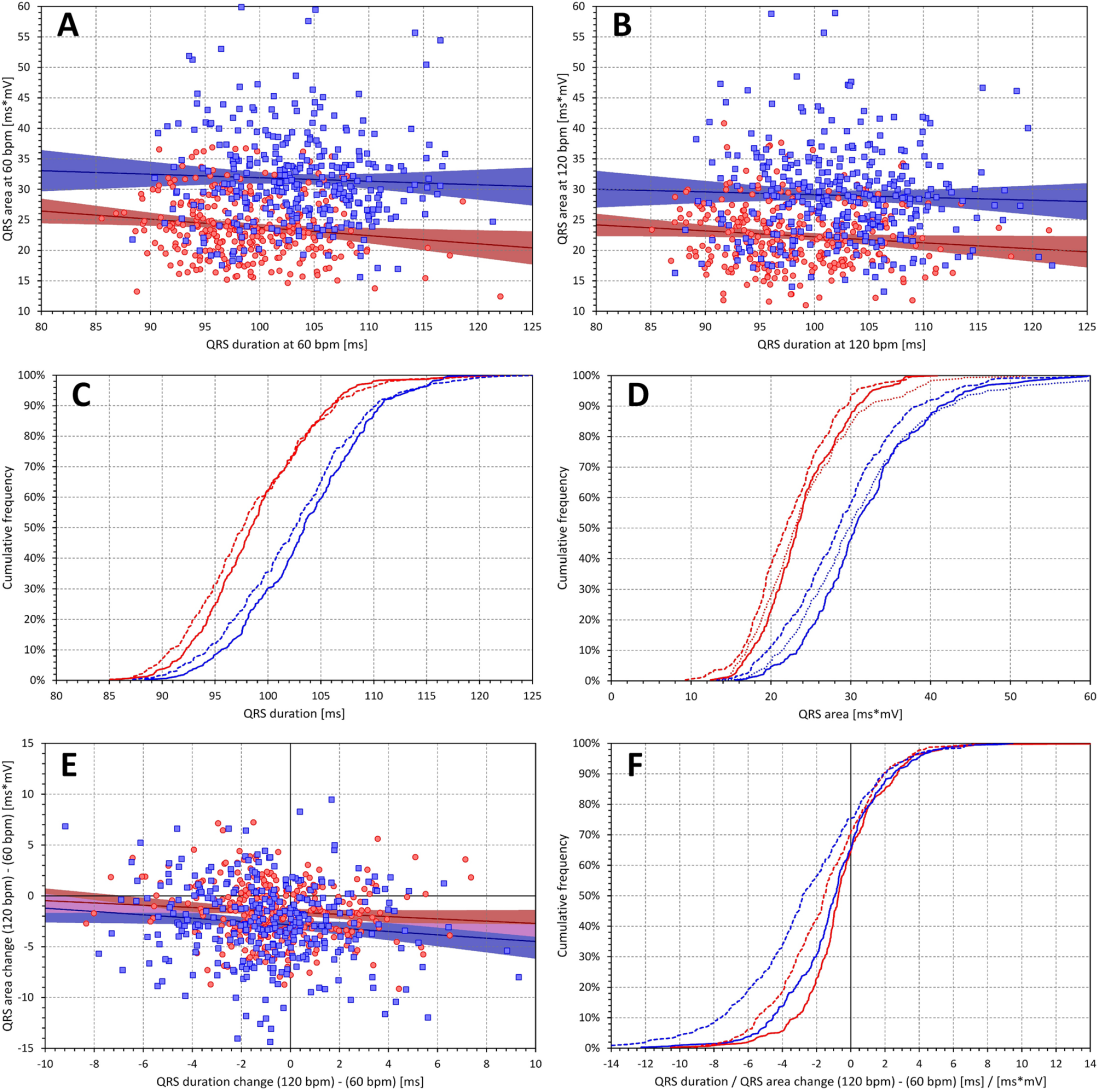
Data of QRS duration and of absolute QRS area. Panels (**A**) and (**B**) show the scatter diagrams of the relationship between the QRS duration and the absolute QRS area at heart rates of 60 and 120 bpm, respectively. Panels (**C**) and (**D**) show the cumulative distributions of QRS duration and of absolute QRS area. Panel (**E**) shows the scatter diagram of the relationship between intra-subject changes of QRS duration and of absolute QRS area corresponding to heart rate changes between 60 and 120 bpm and panel (**F**) shows the cumulative distributions of these intra-subject changes. In panels (**A**), (**B**), and (**E**), the red circles and blue squares show data of female and male subjects, respectively. The solid red and solid blue lines show the linear regressions between the displayed values in females and males, respectively. The red shaded and blue shaded areas are the 95% confidence intervals of the regression lines; the violet areas are the overlaps between the confidence intervals of the sex-specific age-regressions. In panels (**C**), (**D**), and (**F**), the red and blue lines correspond to female and male subjects, respectively. In panels (**C**) and (**D**), the full lines and dashed lines correspond to the values at 60 and 120 bpm, respectively. The dotted lines in panel (**D**) show the values at QRS duration of 100 ms. The full and dashed lines in panel (**F**) show the changes of QRS duration and of absolute QRS area, respectively.

Consistent with previous observations^9^, QRS durations were significantly shorter in females than in males (98.9 ± 5.5 vs 103.5 ± 5.9 ms, p < 0.00001, at 60 bpm, Fig. 6). As also seen in Fig. 6, total QRS area, i.e. the ${{\varvec{\Delta}}}_{0}$ value, was also significantly smaller in females compared to males but this difference was unrelated to the differences in QRS durations (when projected to 100 ms of QRS duration, the ${{\varvec{\Delta}}}_{0}$ areas were 24.21 ± 6.52 and 31.26 ± 9.26 ms*mV in female and males, respectively, p < 0.00001).

Also consistent with previous observations^9^, we observed QRS widening with increasing heart rate in approximately 30% of the subjects while QRS was shortening with increasing heart rate in others. Similar proportions of subjects showed QRS area to increase and decrease with increasing heart rate but the heart rate influences of QRS duration and of QRS area were practically independent of each other (Fig. 6).

### Components of the 3-dimensional space

**Figure 7:**
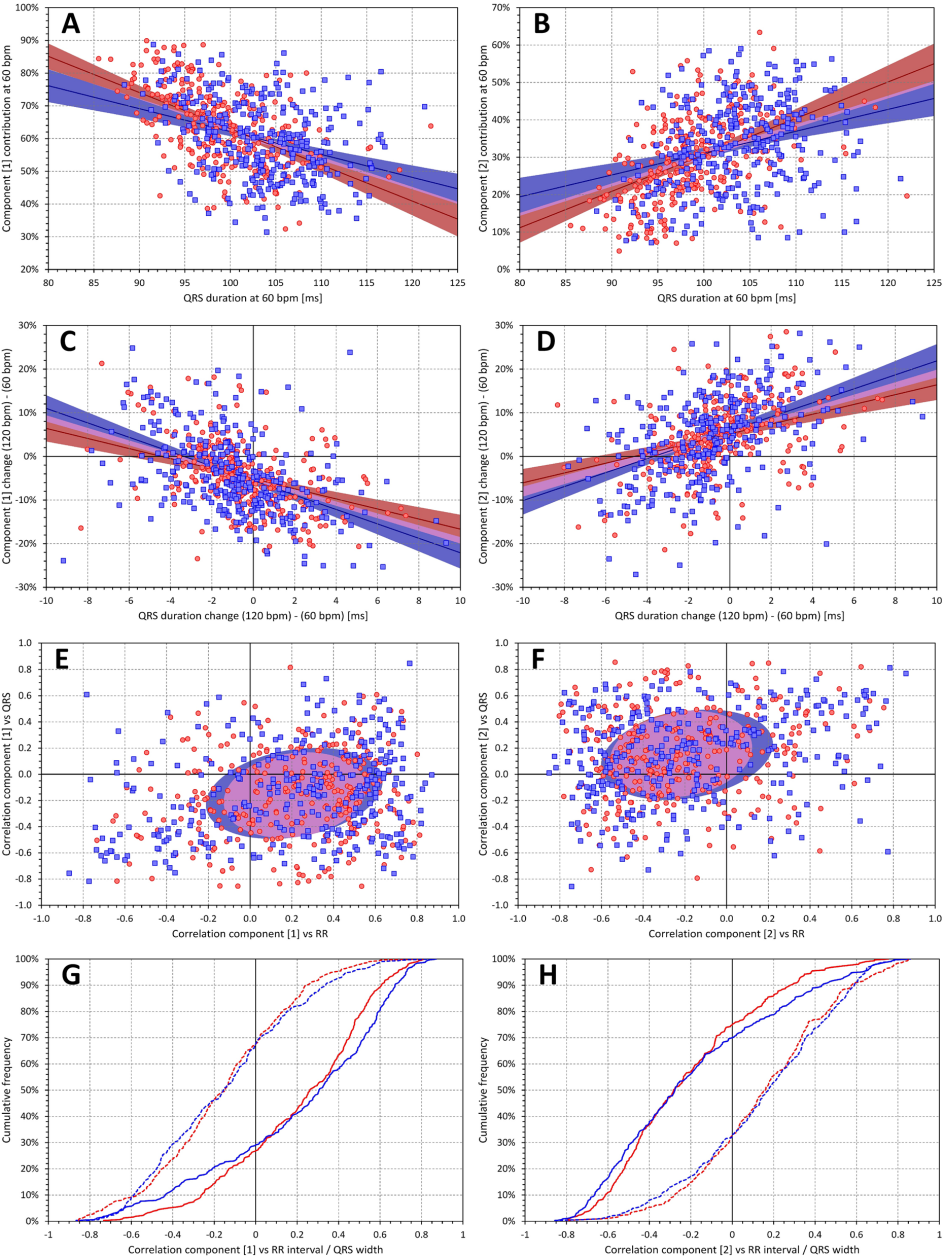
Data of first two components ${\nabla }_{1}$ and ${\nabla }_{2}$. On the left side, panel (**A**) shows the scatter diagram of the relationship of ${\nabla }_{1}$ to QRS duration at heart rate of 60 bpm. Panel (**C**) shows the scatter diagram of the ${\nabla }_{1}$ changes between 120 and 60 bpm to the corresponding changes of QRS duration. Panel (**E**) shows the scatter diagram of the relationship between intra-subject correlation coefficients ${\nabla }_{1}$ versus RR interval and ${\nabla }_{1}$ versus QRS duration. Panel (**G**) shows the cumulative distributions of these correlation coefficients. On the right side, panels (**B**), (**D**), (**F**) and (**H**) show the same data comparisons for ${\nabla }_{2}$ component. The meaning of the symbols in scatter diagrams in panels (**A**), (**B**), (**C**), and (**D**) is the same as in the scatter diagrams of Fig. 6. In panels (**E**) and (**F**), the red circles and blue squares again correspond to the female and male subjects, respectively. The light red and light blue elliptical shapes show the mean ± standard deviation of the correlation coefficients (the elliptical shapes are oriented to represent the optimal orthogonal projection of the data). The violet areas are the overlaps between the sex-specific elliptical shapes. In panels (**G**) and (**H**), the red and blue lines correspond to females and males, respectively; the full and dashed lines show the intra-subject correlation coefficients of the orthogonal components to heart rate and to the QRS duration, respectively.

Within individual subjects, the first relative component (i.e. the ${\nabla }_{1}$ value) was, on average, negatively but moderately correlated with QRS duration (intra-subject Spearman correlation r of − 0.159 ± 0.327 and − 0.146 ± 0.347 in females and males, respectively). It was therefore somewhat surprising that the population correlations between ${\nabla }_{1}$ and QRS duration assessed at specific heart rates were noticeably stronger (− 0.529 and − 0.346 at the rate of 60 bpm, and − 0.528 and − 0.309 at 120 bpm, in females and males, respectively). There was also a noticeable discrepancy between intra-subject relationship of ${\nabla }_{1}$ to QRS duration and to the underlying heart rate. While the intra-subject correlation to QRS was positive in approximately 30% of the subjects, the intra-subject correlation to heart rate was positive in approximately 70% of the subjects. All this is shown in the left panels of Fig. 7 (note the cumulative distributions in panel G of the figure).

The right panels of Fig. 7 show that the relationship of ${\nabla }_{2}$ to heart rate and QRS duration was almost exactly the opposite to that of the ${\nabla }_{1}$. Note that the cumulative distributions shown in panels G and H of the figure are practically mirror images of each other. While the intra-subject correlation with QRS width was only moderately positive (intra-subject r of 0.155 ± 0.330 and 0.149 ± 0.345 in females and males) the population correlation assessed at 60 bpm led to r of 0.474 and 0.290 in females and males, respectively. At 120 bpm, the corresponding r values were 0.443 and 0.234.

**Figure 8:**
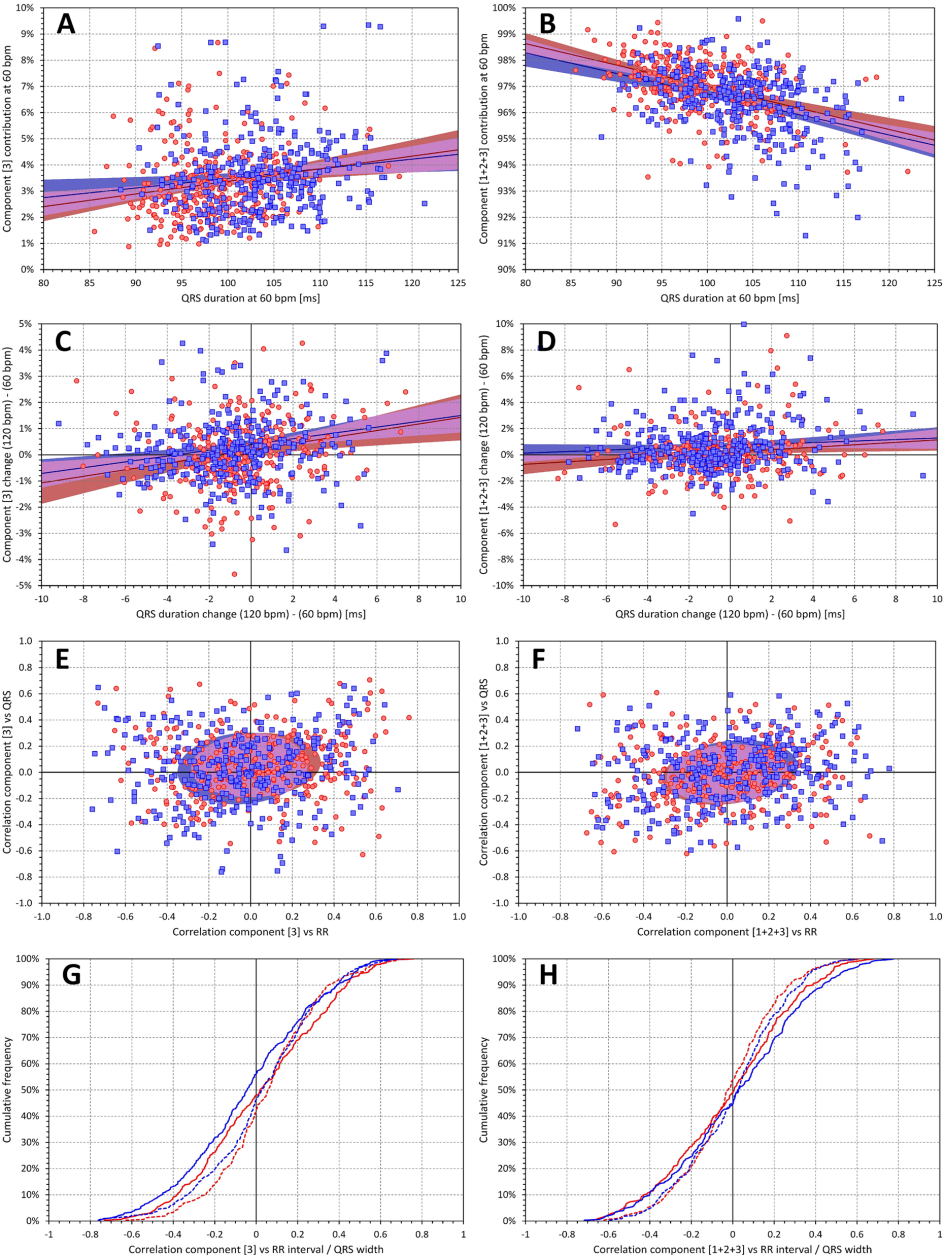
The layout and meaning of panels of the figure is the same as in Fig. 7. The left panels (**A**), (**C**), (**E**), and (**G**) correspond to the component ${\nabla }_{3}$, whilst the right panels (**B**), (**D**), (**F**), and (**H**) correspond to the combination of components ${\nabla }_{1}$+${\nabla }_{2}$+${\nabla }_{3}$.

Left part of Fig. 8 shows that ${\nabla }_{3}$ values were much less related to QRS duration with averages of intra-subject correlations close to 0 and population correlations at 60 bpm with r of 0.188 and 0.140 in females and males, respectively. When all first three components were combined (i.e. values of ${\nabla }_{1}$+${\nabla }_{2}$+${\nabla }_{3}$ considered) more than 90% of the QRS absolute area was covered and the population dependency on QRS duration almost disappeared (right part of Fig. 8).

### QRS fractionation components

**Figure 9:**
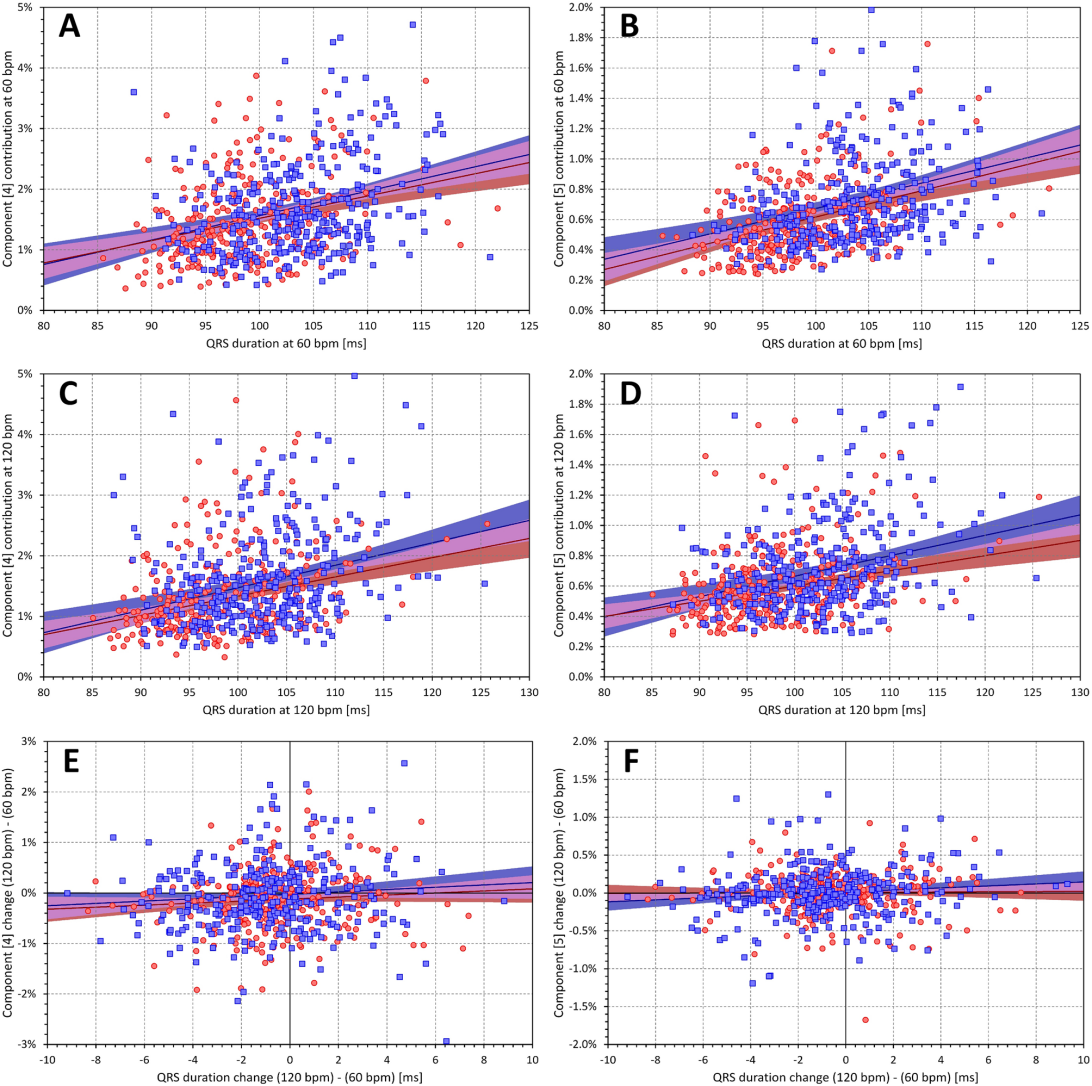
The left panels (**A**) and (**C**) show scatter diagrams of the intra-subject relationship of component ${\nabla }_{4}$ to QRS duration at heart rate of 60 and 120 bpm, respectively. The panel (**E**) shows the scatter diagram of the ${\nabla }_{4}$ changes between 120 and 60 bpm to the corresponding changes of QRS duration. The right panels B, D, and F show the same for component ${\nabla }_{5}$. The meaning of the symbols in scatter diagrams is the same as in the scatter diagrams of Fig. 6.

On average, there were no meaningful intra-subject correlations of ${\nabla }_{4}$, ${\nabla }_{5}$, or ${\nabla }_{6}$ to QRS duration (the absolute values of all r averages were below 0.02). This contrasted with the positive population correlations which, at 60 bpm, led to r values of 0.294 and 0.295 in females and males, respectively, for ${\nabla }_{4}$. For ${\nabla }_{5}$, the corresponding r values were 0.329 and 0.292, while for ${\nabla }_{6}$, the r values were 0.388 and 0.305. Similar gradual increase of r from ${\nabla }_{4}$ to ${\nabla }_{6}$ was also seen at heart rate of 120 bpm (Fig. 9).

**Figure 10:**
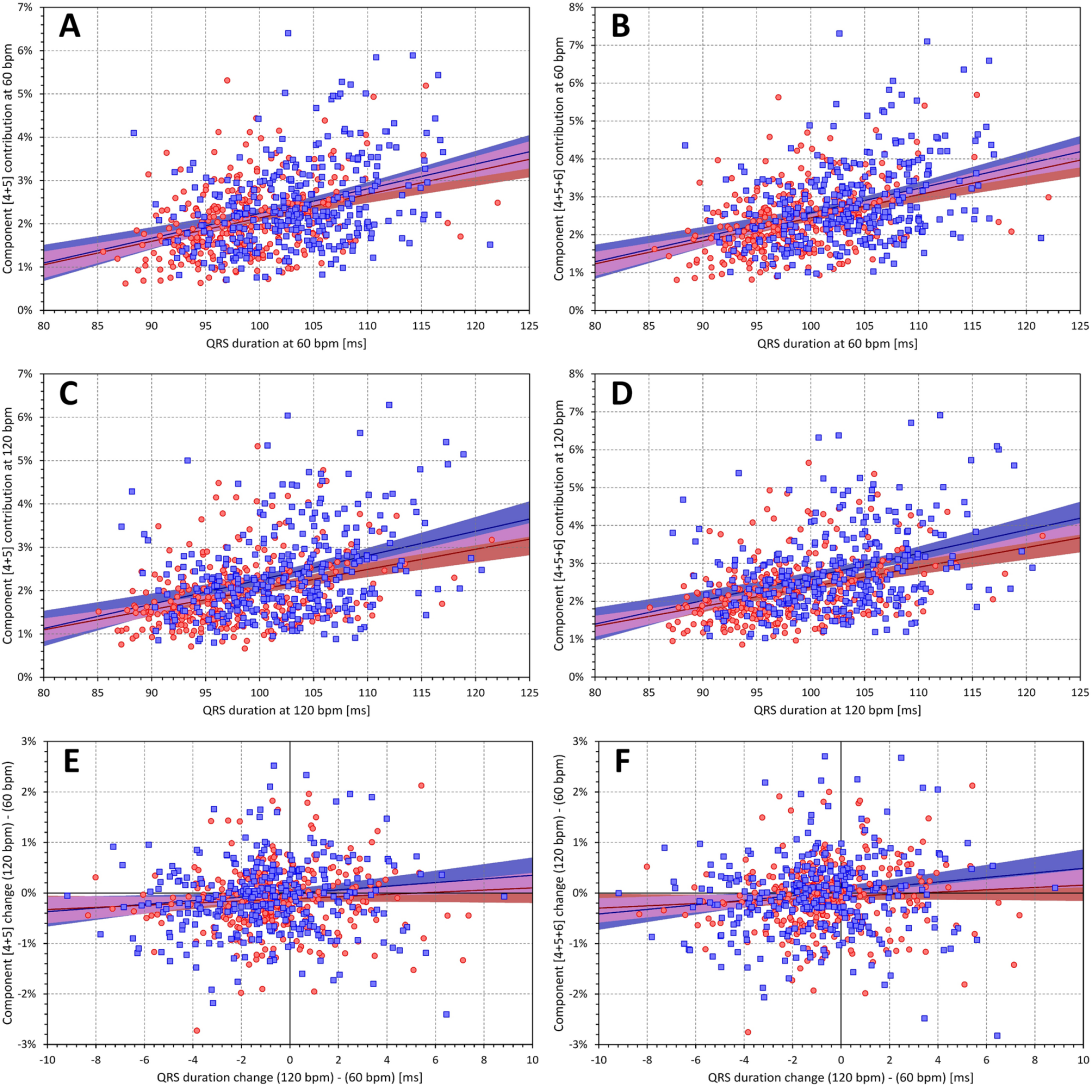
The layout and meaning of panels of the figure is the same as in Fig. 9. The left panels (**A**), (**C**), and (**E**) correspond to the combination of components ${\nabla }_{4}$+${\nabla }_{5}$, whilst the right panels (**B**), (**D**), and (**F**) correspond to the combination of components ${\nabla }_{4}$+${\nabla }_{5}$+${\nabla }_{6}$.

As expected, the contribution of components ${\nabla }_{4}$ to ${\nabla }_{6}$ was gradually decreasing and in the majority of cases, the component combination ${\nabla }_{4}$+${\nabla }_{5}$+${\nabla }_{6}$ represented less than 3% of the absolute QRS area. Compared to ${\nabla }_{4}$+${\nabla }_{5}$, the combination ${\nabla }_{4}$+${\nabla }_{5}$+${\nabla }_{6}$ showed only a small albeit noticeable increase while still maintaining relatively strong correlation with QRS duration (Fig. 10). On the contrary, the population heart rate dependency of components ${\nabla }_{4}$, ${\nabla }_{5}$, and ${\nabla }_{6}$ and of their combinations was practically absent (Figs. 9 and 10).

### Decomposition residuals

**Figure 11:**
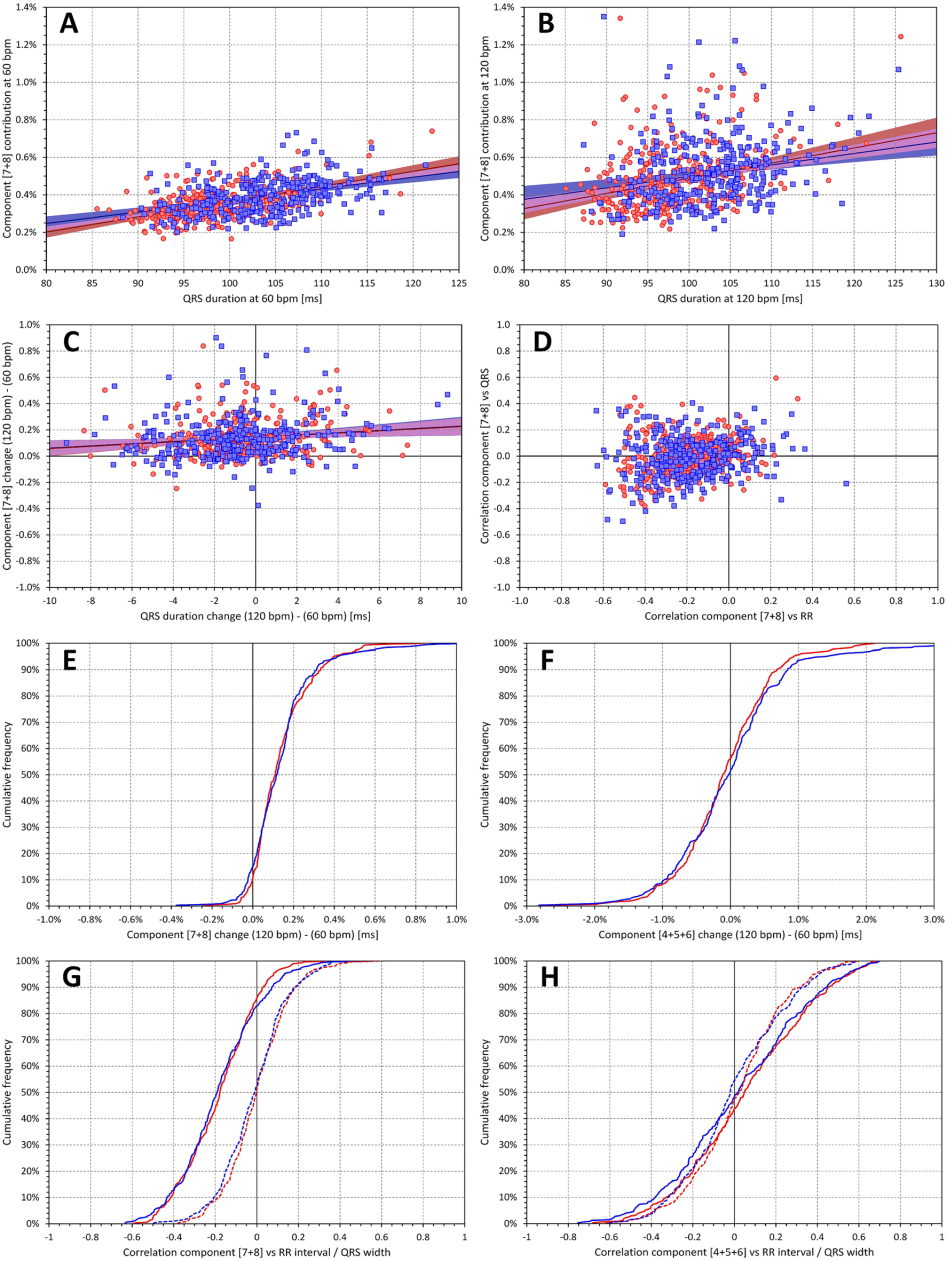
Panels (**A**) and (**B**) show scatter diagrams of the intra-subject relationship of component combination ${\nabla }_{7}$+${\nabla }_{8}$ to QRS duration at heart rate of 60 and 120 bpm, respectively. Panel (**C**) shows the scatter diagram of the ${\nabla }_{7}$+${\nabla }_{8}$ changes between 120 and 60 bpm to the corresponding changes of QRS duration. The meaning of the symbols in these scatter diagrams is the same as in the scatter diagrams of Fig. 6. Panel (**D**) shows the scatter diagram of the relationship between intra-subject correlation coefficients ${\nabla }_{7}$+${\nabla }_{8}$ versus RR interval and ${\nabla }_{7}$+${\nabla }_{8}$ versus QRS duration. The layout of this panel is the same as panels (**E**) and (**F**) in Fig. 7 (note that the data points are clustered so compactly that the underlying elliptical shapes are not easily visible). Panel (**E**) shows the cumulative distribution of intra-subject ${\nabla }_{7}$+${\nabla }_{8}$ changes between heart rates of 120 and 60 bpm; for comparison, panel (**F**) shows the same for ${\nabla }_{4}$+${\nabla }_{5}$+${\nabla }_{6}$ changes. Panel (**G**) shows the cumulative distribution of intra-subject correlations of ${\nabla }_{7}$+${\nabla }_{8}$ versus RR interval (full lines) and versus QRS duration (dashed lines). For comparison, panel (**H**) shows the same for intra-subject ${\nabla }_{4}$+${\nabla }_{5}$+${\nabla }_{6}$ correlations. In panels (**E**), (**F**), (**G**), and (**H**), the red and blue lines correspond to female and male subjects, respectively.

The situation was different with the combination of final components ${\nabla }_{7}$+${\nabla }_{8}$(Fig. 11). On average, this combination showed no intra-subject correlation with QRS complex (r of 0.001 ± 0.151 and − 0.014 ± 0.157 for females and males, respectively). However, in population data, significant correlations with QRS complex were observed. At the rate of 60 bpm, r values of 0.521 and 0.409 were observed for females and males, respectively. At 120 bpm, these r values decreased to 0.332 and 0.237, respectively. Substantial intra-subject effect of heart rate was also noted. Although numerically rather small, the value ${\nabla }_{7}$+${\nabla }_{8}$ increased from 0.364 ± 0.087 and 0.402 ± 0.093% in females and males at 60 bpm, to 0.501 ± 0.172 and 0.538 ± 0.189% at 120 bpm (p < 0.00001 and p = 0.012 in females and males, respectively).

### Sex comparisons

**Figure 12:**
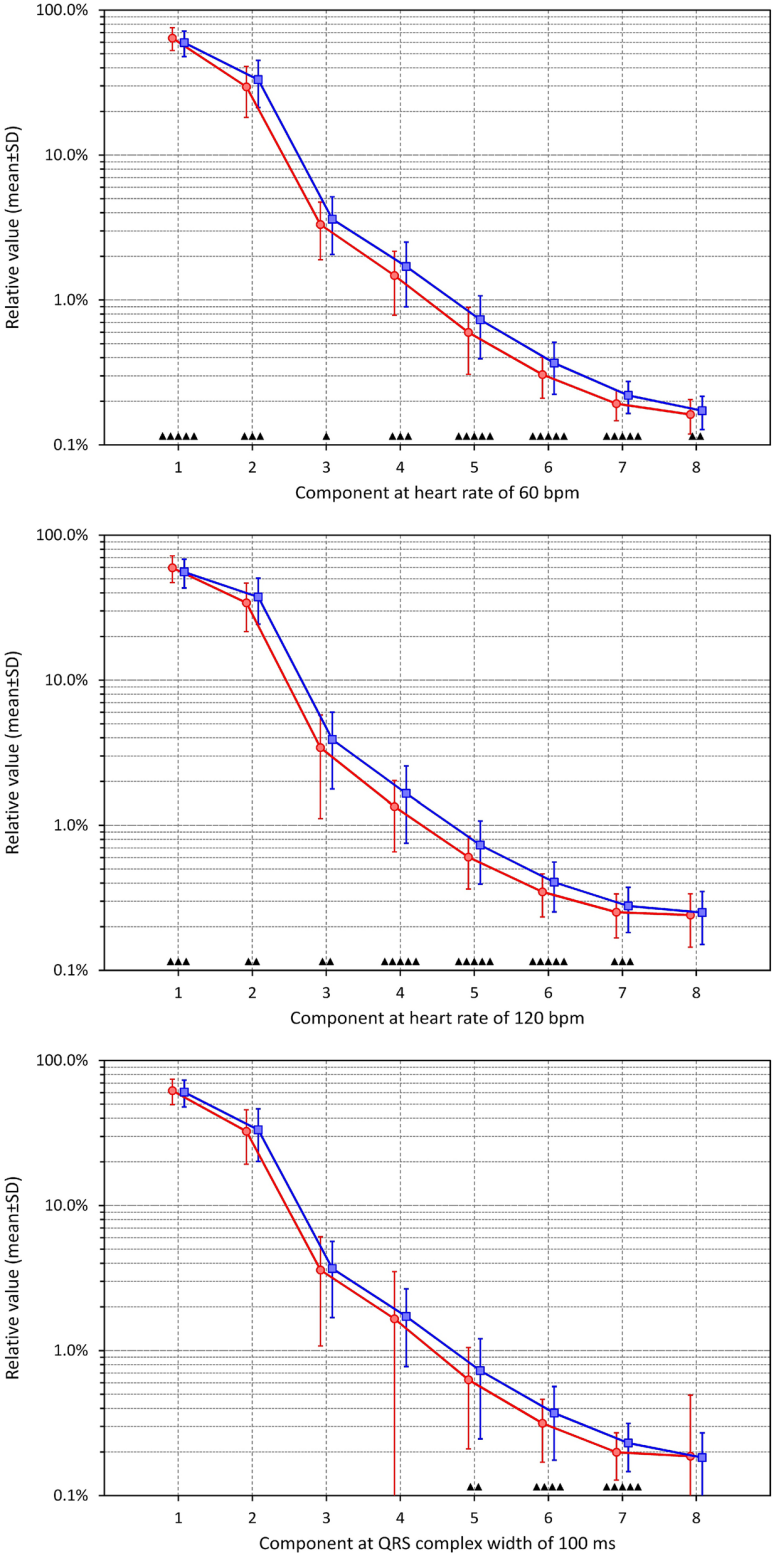
The panels show the mean (± standard deviation) values of decomposition components ${\nabla }_{1}$ to ${\nabla }_{8}$ for female (red graphs) and male (blue graphs) subjects. The top, middle, and bottom panels show the values of components assessed at 60 bpm heart rate, 120 bpm heart rate, and 100 ms of QRS duration, respectively. For each component showing statistically significant difference between females and males, the level of significance is shown above the horizontal axis (▲—p < 0.05, ▲▲—p < 0.01, ▲▲▲—p < 0.001, ▲▲▲▲—p < 0.0001, ▲▲▲▲▲—p < 0.00001). Note the logarithmic scale of the vertical axes.

Since these observations suggest the electrophysiologic basis for shorter QRS duration in females compared to males, Fig. 12 summarises the observed sex differences.

The results were similar for components values projected to 60 bpm and to 120 bpm. Females showed larger ${\nabla }_{1}$ compared to males (e.g. 64.2 ± 11.6 vs 59.7 ± 11.9% at 60 bpm, p < 0.00001) while all ${\nabla }_{2}$ to ${\nabla }_{7}$ are larger in males with different levels of statistical significance. ${\nabla }_{8}$ were also larger in males than in females but we observed statistically significant differences only at 60 bpm while at 120 bpm, the values were still numerically smaller in females (0.241 ± 0.096 vs 0.250 ± 0.099%) but not significantly different.

When eliminating the influence of different QRS durations by using data projected to the same level of 100 ms, the sex comparison was different. The components ${\nabla }_{1}$ to ${\nabla }_{3}$ were practically the same between sexes. ${\nabla }_{4}$ to ${\nabla }_{7}$ were larger in males than in females (the difference did not reach statistical significance for ${\nabla }_{4}$, likely because large spread of projected values in females) while ${\nabla }_{8}$ showed again no difference between sexes.

## Discussion

In addition to the setup of the technology, these data analyses offer electrophysiological insights and lead to potentially unexpected observations.

Previous clinical experience with vectorcardiography^32–34^ might lead to expectations that the first two (if not the first three) components ${\nabla }_{1}$ to ${\nabla }_{2}$ (or, in some cases, to ${\nabla }_{3}$) would contribute similarly to the 3-dimensional composite of the QRS complex. Indeed, the directions of the projections of the first 3 components also create an orthogonal 3-dimensional system which can be represented as an optimal rotation of the XYZ coordinate system so that the maximum QRS power projects into the first direction, the maximum of the reminder into the perpendicular second direction, and so on. Nevertheless, the optimum orientation of coordinates by SVD results in different proportions of the components. It is easy to understand that, in principle, ${\nabla }_{3}$ is a measure of QRS planarity^33,35^. Since it is known that electrophysiology of normal hearts is characterised by an almost perfect planarity of the spatial QRS loop^36,37^, the contribution of ${\nabla }_{3}$ only around 3% to 4% of the total QRS absolute area is not surprising. Nevertheless, the practically 2:1 proportion between ${\nabla }_{1}$ to ${\nabla }_{2}$ was somewhat unexpected as was the proportion of subjects in whom ${\nabla }_{1}$ exceeded 80% and ${\nabla }_{2}$ was below 20% (see top panels of Fig. 7). Although similar proportion has previously been described for the eigenvalues of resting ECGs^23^ we initially assumed that similar to the vectorcardiography images, the contributions of ${\nabla }_{1}$ and ${\nabla }_{2}$ would not be this different since the proportions of this decomposition are not similar those of eigenvalues (see Fig. 5).

The marked differences between the intra-subject and inter-subject relationship of the components to the QRS complex duration suggest that the proportions of the components (perhaps except for ${\nabla }_{7}$ and ${\nabla }_{8}$) are not determined dynamically by physiologic regulatory processes but are driven by individual anatomic and histologic composites of the ventricular myocardium. Our observations also strongly support the physiologic expectation that even in healthy subjects, narrower QRS complex is a consequence of a more direct and less convoluted path of the depolarisation sequence.

Although the ECG signals were not only filtered but also processed by the construction of median representative beat (which, we believe, also has noise-reduction properties) before applying the SVD analysis, the component ${\nabla }_{8}$, and perhaps also the component ${\nabla }_{7}$, represents mainly the residual noise in the recordings. This is consistent with the marked increase of these components between rates of 60 and 120 bpm which is frequently accompanied by some decrease of signal quality especially if the heart rate increase reflects physical activity.

The observation of the likely relationship between QRS duration and the convolutedness of the depolarisation sequence might also provide insight into the sex difference of QRS complex. The shorter QRS complex duration in females has previously been repeatedly described^38^. It has also been repeatedly proposed that this sex difference might reflect differences in heart sizes. Nevertheless, this has not been confirmed^9^. Rather, the sex differences might be driven by the depolarisation sequence initiated by the His-Purkinje system. Our results seem to indicate that compared to males, the propagation of the depolarisation sequence in females is more direct and less curled and twisted. This is not supported only by the proportions between ${\nabla }_{1}$ (larger in females) and ${\nabla }_{2}$ (larger in males) but also by the sex differences in the higher components above the 3-dimensional projections as shown in Fig. 12. Such a lesser complexity of the female depolarisation sequence might also be the basis for the known decreased incidence of ventricular tachyarrhythmias in females^39–41^ (with the exception of channelopathy-based arrhythmias^42^ that are unrelated to the depolarisation abnormalities). Since the present study evaluated healthy adults with females largely of pre-menopausal ages, we cannot comment on whether the observed sex differences are a consequence of hormonal influence. Electrocardiography studies in children and adolescents^43^ as well as studies of the influence of menstrual cycle are needed to address these details.

SVD analyses have previously been applied also to body surface maps and multi-lead ECGs^44,45^. These analyses showed that larger number of independent ECG components can be detected. Nevertheless, since we had only 12-lead ECG available, we were unable to decompose the signals to more than 8 algebraic dimensions corresponding to the number of independent leads. While a system analysing standard 10-s 12-lead ECGs is more practical for further applications of the technology that we have described here, application of the same principles to multi-lead ECG recordings would be of research interest.

Visible fragmentation of the QRS complex has repeatedly been described as an indicator of increased risk of adverse cardiac events^46–49^. It has also been observed that fragmented QRS complex is more often present in males compared to females^50^ and that, in cardiac patients, QRS abnormalities were more predictive in patients with prolonged QRS complex^51^. Having used data of healthy subjects, we cannot link our methodological results to these studies directly. Nevertheless, the logic of the analysis suggests that the comparison between the orthogonal components ${\nabla }_{1}$ to ${\nabla }_{3}$ and the value of the ${\nabla }_{4}$ to ${\nabla }_{6}$ (or perhaps ${\nabla }_{4}$ to ${\nabla }_{7}$) components might offer automatic detection of fragmented QRS complex including the distinction of micro-fragmented cases that are not directly apparent during standard visual judgement and diagnosis. Further studies are needed in this respect, but the data presented here might suggest prospectively defined normality limits (e.g. 3.5% for the combination of ${\nabla }_{4}$+${\nabla }_{5}$+${\nabla }_{6}$ – see Fig. 10). The so-called non-dipolar components (based on eigenvalue proportions) of the T wave have previously been proposed as risk indicators in cardiac patients^52^. It seems therefore logical to extend this technology also to repolarisation signals.

QRS complex duration, its morphological characteristics, and ECG-based depolarisation abnormalities are also used to optimise and to predict the outcome of cardiac resynchronisation therapy^53,54^. We are again unable to comment on the possible use of the described technology for these purposes but, based on the electrophysiologic understanding of the analysis, it appears feasible to suggest that studies of this kind might be worth conducting.

Intentionally, we analysed only ECG segments preceded by stable heart rate. This allowed avoiding potential problems with heart rate hysteresis and led to inter-subject differences in the number of analysed ECG segments. Hysteresis-driven disparity between ECG indices and simultaneously measured heart rate occurs when the adaptation to heart rate changes is delayed. This is a known property of repolarisation-related intervals^55–57^ which has important practical implications^58,59^. Nevertheless, it is not known whether QRS duration exhibits any delayed heart rate adaptation^9^. Similarly, based on the analysed data, we cannot address the question of how quickly the decomposition components alter in response to abrupt heart rate changes.

### Limitations

Further limitations of the analytical technique also need to be considered. The algebraic process of SVD is based on signal reconstruction analysis and therefore cannot map the depolarisation wave propagation directly. The ECG images of localised depolarisation abnormalities might cancel each other and thus be beyond SVD detection. The presented analysis might therefore underestimate but not overestimate depolarisation abnormalities. As already stated, while the span of heart rates covered by different ECGs in individual subjects was reasonably wide, the span of the QRS durations was limited in some subjects. In such cases, the regression projections to 100 ms of QRS duration might have involved substantial extrapolation beyond available data and might thus have polluted the estimates by some errors. The source clinical studies did not involve echocardiographic examinations and we are thus unable to relate the observations to anatomical heart sizes. The original Holter recordings used Mason-Likar electrode positions. We cannot answer the question of whether this influenced the measured values and whether any derived normality limits would be different if standard ECG leads were used. The spans of the ages of the subjects were not wide enough to investigate age influence. Although the ECG signal quality might be influenced by obesity, we have not related the measurements to body mass index.

## Conclusion

Despite these limitations, the presented technology and data analyses suggest that components of QRS complex beyond the 3-dimensional reconstruction can reliably be estimated and that they have an electrophysiologic role in judging the propagation of the depolarisation waveform through the myocardium. It appears that the detailed convolution of the depolarisation waveform is individual, and that smoother and less intricate depolarisation propagation is the mechanism responsible for shorter QRS duration in females.

## Supplementary Information

Supplementary Information.
