# Supplementary material for: Spatial distribution of physiologic 12-lead QRS complex
Source: Sci Rep. 2021 Feb 22;11:4289. doi: 10.1038/s41598-021-83378-8 (PMC7900243; doi:10.1038/s41598-021-83378-8)
Supplement: Supplementary file 1 — Supplementary Information. [file 41598_2021_83378_MOESM1_ESM.docx]

# **Spatial distribution of physiologic 12-lead QRS complex**

by

Katerina Hnatkova, Irena Andršová, Ondřej Toman, Peter Smetana, Katharina M Huster,
Martina Šišáková, Petra Barthel, Tomáš Novotný, Georg Schmidt, Marek Malik

# **Supplementary table of measured values**

|  |  | Females | Males | p-value |
| --- | --- | --- | --- | --- |
| QRS Area [ms*mV] | RR slope | 0.104 ± 0.187 | 0.137 ± 0.201 | 0.031 |
|  | RR correlation | 0.163 ± 0.322 | 0.278 ± 0.333 |  |
|  | QRS slope | 0.958 ± 2.055 | 1.242 ± 2.007 | NS |
|  | QRS correlation | 0.132 ± 0.264 | 0.214 ± 0.273 |  |
|  | 60 bpm projection | 23.90 ± 5.11 | 31.70 ± 7.55 | <0.00001 |
|  | 120 bpm projection | 22.36 ± 5.27 | 29.01 ± 7.61 | <0.00001 |
|  | 100 ms projection | 24.21 ± 6.52 | 31.26 ± 9.26 | <0.00001 |
| QRS [ms] | RR slope | 0.008 ± 0.037 | 0.014 ± 0.040 | 0.038 |
|  | RR correlation | 0.106 ± 0.327 | 0.140 ± 0.340 |  |
|  | 60 bpm projection | 98.9 ± 5.5 | 103.5 ± 5.9 | <0.00001 |
|  | 120 bpm projection | 98.4 ± 6.2 | 102.5 ± 6.5 | <0.00001 |
| $\boldsymbol{\nabla}_{1}$ [%] | RR slope | 0.114 ± 0.208 | 0.107 ± 0.273 | NS |
|  | RR correlation | 0.214 ± 0.340 | 0.212 ± 0.423 |  |
|  | QRS slope | -1.048 ± 2.120 | -1.104 ± 2.179 | NS |
|  | QRS correlation | -0.159 ± 0.327 | -0.146 ± 0.347 |  |
|  | 60 bpm projection | 64.2 ± 11.6 | 59.7 ± 11.9 | <0.00001 |
|  | 120 bpm projection | 59.6 ± 12.5 | 55.7 ± 12.6 | <0.001 |
|  | 100 ms projection | 62.0 ± 12.3 | 60.5 ± 12.7 | NS |
| $\boldsymbol{\nabla}_{2}$ [%] | RR slope | -0.215 ± 0.357 | -0.184 ± 0.422 | NS |
|  | RR correlation | -0.218 ± 0.341 | -0.190 ± 0.412 |  |
|  | QRS slope | 1.829 ± 4.270 | 1.582 ± 4.159 | NS |
|  | QRS correlation | 0.155 ± 0.330 | 0.149 ± 0.345 |  |
|  | 60 bpm projection | 29.6 ± 11.4 | 33.1 ± 11.9 | <0.001 |
|  | 120 bpm projection | 34.2 ± 12.5 | 37.4 ± 13.1 | 0.001 |
|  | 100 ms projection | 32.5 ± 13.2 | 33.3 ± 13.1 | NS |
| $\boldsymbol{\nabla}_{3}$ [%] | RR slope | -0.002 ± 0.588 | -0.066 ± 0.482 | NS |
|  | RR correlation | 0.026 ± 0.310 | -0.039 ± 0.311 |  |
|  | QRS slope | 1.016 ± 5.964 | 0.206 ± 5.282 | NS |
|  | QRS correlation | 0.055 ± 0.246 | 0.029 ± 0.274 |  |
|  | 60 bpm projection | 3.32 ± 1.42 | 3.61 ± 1.55 | 0.013 |
|  | 120 bpm projection | 3.43 ± 2.32 | 3.89 ± 2.12 | 0.009 |
|  | 100 ms projection | 3.59 ± 2.52 | 3.68 ± 1.99 | NS |
|  |  | Females | Males | p-value |
| $\boldsymbol{\nabla}_{4}$ [%] | RR slope | 0.142 ± 0.595 | 0.062 ± 0.560 | NS |
|  | RR correlation | 0.077 ± 0.290 | 0.038 ± 0.319 |  |
|  | QRS slope | 0.842 ± 6.53 | -0.097 ± 5.28 | 0.047 |
|  | QRS correlation | 0.020 ± 0.216 | -0.013 ± 0.246 |  |
|  | 60 bpm projection | 1.48 ± 0.69 | 1.70 ± 0.81 | <0.001 |
|  | 120 bpm projection | 1.35 ± 0.69 | 1.66 ± 0.90 | <0.00001 |
|  | 100 ms projection | 1.66 ± 1.84 | 1.72 ± 0.94 | NS |
| $\boldsymbol{\nabla}_{5}$ [%] | RR slope | -0.045 ± 0.568 | 0.005 ± 0.564 | NS |
|  | RR correlation | -0.012 ± 0.236 | -0.007 ± 0.252 |  |
|  | QRS slope | -0.106 ± 5.461 | 0.429 ± 5.641 | NS |
|  | QRS correlation | -0.004 ± 0.166 | 0.024 ± 0.199 |  |
|  | 60 bpm projection | 0.598 ± 0.291 | 0.731 ± 0.338 | <0.00001 |
|  | 120 bpm projection | 0.604 ± 0.241 | 0.732 ± 0.339 | <0.00001 |
|  | 100 ms projection | 0.629 ± 0.419 | 0.727 ± 0.481 | 0.006 |
| $\boldsymbol{\nabla}_{6}$ [%] | RR slope | -0.185 ± 0.485 | -0.143 ± 0.500 | NS |
|  | RR correlation | -0.065 ± 0.187 | -0.058 ± 0.210 |  |
|  | QRS slope | -0.228 ± 4.722 | -0.131 ± 4.574 | NS |
|  | QRS correlation | -0.001 ± 0.142 | -0.002 ± 0.158 |  |
|  | 60 bpm projection | 0.306 ± 0.096 | 0.367 ± 0.144 | <0.00001 |
|  | 120 bpm projection | 0.348 ± 0.115 | 0.406 ± 0.153 | <0.00001 |
|  | 100 ms projection | 0.316 ± 0.146 | 0.370 ± 0.195 | <0.0001 |
| $\boldsymbol{\nabla}_{7}$ [%] | RR slope | -0.353 ± 0.403 | -0.306 ± 0.453 | NS |
|  | RR correlation | -0.127 ± 0.153 | -0.124 ± 0.178 |  |
|  | QRS slope | -0.333 ± 4.434 | -0.281 ± 4.405 | NS |
|  | QRS correlation | -0.001 ± 0.127 | -0.011 ± 0.137 |  |
|  | 60 bpm projection | 0.193 ± 0.046 | 0.220 ± 0.055 | <0.00001 |
|  | 120 bpm projection | 0.252 ± 0.084 | 0.278 ± 0.096 | <0.001 |
|  | 100 ms projection | 0.199 ± 0.071 | 0.230 ± 0.084 | <0.00001 |
| $\boldsymbol{\nabla}_{8}$ [%] | RR slope | -0.497 ± 0.592 | -0.485 ± 0.451 | NS |
|  | RR correlation | -0.192 ± 0.140 | -0.192 ± 0.159 |  |
|  | QRS slope | -0.197 ± 7.666 | -0.056 ± 6.216 | NS |
|  | QRS correlation | 0.004 ± 0.141 | -0.010 ± 0.135 |  |
|  | 60 bpm projection | 0.162 ± 0.043 | 0.172 ± 0.045 | 0.004 |
|  | 120 bpm projection | 0.241 ± 0.096 | 0.250 ± 0.099 | NS |
|  | 100 ms projection | 0.187 ± 0.305 | 0.183 ± 0.088 | NS |

For the total QRS area (average of all 8 independent ECG leads), QRS duration, and each of the de­composition components, the table shows the slopes of the log-linear regressions to the RR intervals of the underlying heart rate and to the QRS durations, corresponding Spearman correlation coeffi­cients, and the regression-based projections to the heart rate of 60 and 120 beats per minute (bpm) and to the QRS duration of 100 ms. The values shown are mean ± standard deviation displayed sepa­rately for female and male subjects. The log-linear regression slopes and the regression projected val­ues were compared between females and males using two-sample, two-tail t-test assuming different standard deviations of compared samples.
